# Solvent-Free Patterning of Colloidal Quantum Dot Films Utilizing Shape Memory Polymers

**DOI:** 10.3390/mi8010018

**Published:** 2017-01-10

**Authors:** Hohyun Keum, Yiran Jiang, Jun Kyu Park, Joseph C. Flanagan, Moonsub Shim, Seok Kim

**Affiliations:** 1 Department of Mechanical Science and Engineering, University of Illinois at Urbana–Champaign, Urbana, IL 61801, USA; hkeum2@illinois.edu (H.K.); jpark323@illinois.edu (J.K.P.); 2 Department of Material Science and Engineering, University of Illinois at Urbana–Champaign, Urbana, IL 61801, USA; yjiang40@illinois.edu (Y.J.); jcflana2@illinois.edu (J.C.F.); mshim@illinois.edu (M.S.)

**Keywords:** colloidal quantum dots, shape memory polymer, patterning

## Abstract

Colloidal quantum dots (QDs) with properties that can be tuned by size, shape, and composition are promising for the next generation of photonic and electronic devices. However, utilization of these materials in such devices is hindered by the limited compatibility of established semiconductor processing techniques. In this context, patterning of QD films formed from colloidal solutions is a critical challenge and alternative methods are currently being developed for the broader adoption of colloidal QDs in functional devices. Here, we present a solvent-free approach to patterning QD films by utilizing a shape memory polymer (SMP). The high pull-off force of the SMP below glass transition temperature (*T*_g_) in conjunction with the conformal contact at elevated temperatures (above *T*_g_) enables large-area, rate-independent, fine patterning while preserving desired properties of QDs.

## 1. Introduction

The variable size, shape, and composition of colloidal semiconductor quantum dots (QDs) permit convenient tailoring of electrical and optical properties [1,2,3,4]. In addition, solubility of colloidal QDs in a variety of solvents can be beneficial to low-cost, large-area manufacturing compared to conventional micro-manufacturing [5]. In particular, the use of solution-based fabrication techniques [6,7,8] has attracted much attention for next-generation devices from colloidal QDs in the fields of light-emitting diodes (LEDs) [9,10,11], solar cells [12,13,14,15], and thin film transistors (TFTs) [8,16]. While solubility allows for convenient spin-casting of thin films of QDs, further processing of the deposited films is limited due to solvent stripping/damage that can easily occur, which then hinders the broader adoption of QDs for such photonic and electronic devices. In this context, direct patterning of QD through both additive and subtractive means has been studied. Additive patterning schemes such as dip-pen and polymer pen nanolithography have successfully demonstrated patterning of a single QD resolution [17,18,19]. On the other hand, subtractive patterning of QD films through transfer printing-based mechanical peeling using elastomeric stamps has been investigated and high-quality transferred films have recently been demonstrated [20,21,22,23].

Transfer printing with a structured polydimethylsiloxane (PDMS) elastomeric stamp requires certain thresholds with respect to the applied preload when the PDMS stamp is making a contact with a QD film and with respect to separation rate, i.e., peeling rate, for proper patterning [20,21]. This method suffers from inconsistent patterning due to weak adhesion between the QDs, high preload, a high peeling rate, and poor resolution. Introducing water-soluble polyvinyl alcohol (PVA) can enhance transfer of QD films to PDMS stamps and improve patterning yields, but the process still suffers from high preload, high peeling rate, and moderate resolution [22]. Recently introduced intaglio transfer printing has successfully demonstrated ultra high resolution, but the drawback of the high preload and high peeling rate (10 cm/s) still remains [23].

We propose an alternative method of patterning QD films that utilizes the shape memory effect of shape memory polymers (SMPs) to alleviate such high preload and high peeling rate requirements without compromising high pattern resolution. SMPs are a class of thermosensitive materials that change their mechanical compliance across the polymer’s glass transition or melting temperature (*T*_g_, or *T*_m_) [24,25]. The reversibility in the elastic modulus can be exploited to provide conformal contact and high pull-off force, useful for transfer printing and patterning [26]. Here, a structured SMP surface is used to make conformal contact to QD films with low preload at elevated temperature and to provide high pull-off forces enabling high-resolution large-area patterning, even with a low separation rate (~2 µm/s) at room temperature. In such a way, solvent-free, rate-independent, high yield, highly scalable QD film patterning is achieved via the SMP surface.

## 2. Materials and Methods

### 2.1. Synthesis of CdSe/CdS Core/Shell Quantum Dots

CdSe/CdS core/shell QDs were synthesized by an established method with slight modifications [27]. Briefly, 1 mmol of CdO, 4 mmol of oleic acid (OA), and 20 mL of 1-octadecene (ODE) were degassed at 100 °C for 1 h before being heated up to 300 °C to form clear Cd(OA)_2_ precursors. Then 0.25 mL of 1 M Se in trioctylphosphine was swiftly injected into the reaction mixture. After 90 s of growth, 0.75 mmol of 1-octanethiol in 2 mL of ODE was injected dropwise. The reaction was terminated by cooling with an air jet after another 30 min of growth. The final reaction mixture was purified twice by adding 1 part toluene and 2 parts ethanol and centrifuging at 2000 rpm.

### 2.2. Preparation of Si–ODTS–QD Substrate

**Figure 1:**
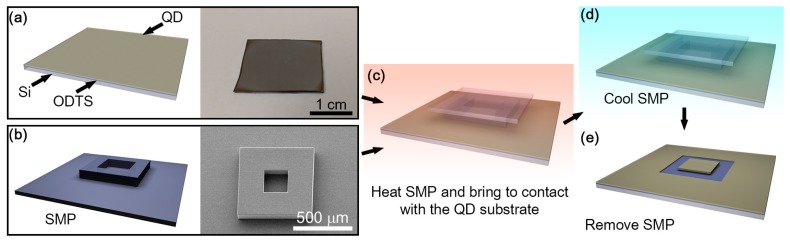
A schematic of the process flow of QD patterning using an SMP stamp. (**a**) An ODTS and QD coated Si substrate is prepared. A photograph of the donor substrate is shown on right. (**b**) An SMP stamp is prepared separately. An scanning electron microscope (SEM) image of the stamp is shown in right. (**c**) The SMP stamp is heated above *T*_g_ and brought in conformal contact with the QD substrate with minimal preload. (**d**) Prior to the separation of the SMP stamp and the QD substrate, the stamp is cooled below *T*_g_ for higher pull-off force. (**e**) QDs are removed at the region where the SMP stamp is contacting the QD substrate, resulting in successful patterning.

The preparation of octadecyltrichlorosilane (ODTS)-coated Si substrates followed an established method [21] with slight modifications. Silicon substrates were cleaned by acetone/isopropanol and dried with N_2_ flow, and then cleaned using UV/O_3_ for 30 min. After UV/O_3_ exposure, the Si substrates were immediately transferred to ODTS in anhydrous hexane solution (1:600 volume ratio) and left to self-assemble for 1 h. The resulting substrates were sonicated in chloroform to remove excess ODTS molecules and then baked on a hot plate at 120 °C for 20 min. Once the ODTS-coated Si substrate was prepared, a solution of QDs in octane (~60 mg/mL) was spin-casted onto the substrate at 2000 rpm for 30 s. The schematics and an optical image of the prepared substrate are included in Figure 1a.

### 2.3. Preparation of the Shape Memory Polymer (SMP) Stamp

For the fabrication of SMP stamps, a SU-8 (SU-8 50, MicroChem, Westborough, MA, USA) mold was first made. A Si wafer was thoroughly degreased with acetone and isopropyl alcohol. Then, the Si wafer was treated with oxygen plasma for further cleaning. SU-8 was then spin-coated to form a 50 µm thin layer on the Si wafer. After a soft baking process on a hotplate at 65 °C for 6 min and at 95 °C for 20 min, the SU-8 layer was patterned through UV exposure with a pre-designed FeO_2_ mask. Post exposure baking on a hotplate at 65 °C for 1 min and 95 °C for 5 min and developing with SU-8 developer resulted in a 50-µm-thick SU-8 mold. For the double molding process, a SU-8 mold on a Si wafer was coated with trichloro-1*H*,1*H*,2*H*,2*H*-perfluorodecylsilane (FDTS) through molecular vapor deposition (MVD) to form an anti-stick monolayer. After fully mixing and degassing, a PDMS precursor was carefully poured into a SU-8 mold and cured in a convection oven at 60 °C for 120 min. The PDMS mold was then used for the successive SMP molding process. A previously developed SMP, NGDE2 with *T*_g_ of ~40 °C [28], was used in this work. A mixed SMP precursor was poured into the PDMS mold and cured between the mold and a glass slide in a convection oven at 100 °C for 90 min. Final demolding of the SMP from the PDMS mold leads to a SMP stamp 50 µm in thickness on the glass slide as shown in Figure 1b.

### 2.4. Patterning Procedure

On a high precision translational and rotational mechanical stage, the prepared Si–ODTS–QD substrate is placed. The SMP stamp is attached to an indium tin oxide (ITO) heater designed to generate heat to increase temperature to 100 °C upon 16 V bias by an external power source. This ITO heater with the SMP stamp is attached to a fixture, which is placed over the Si–ODTS–QD substrate. An optical microscope is located over the ITO heater, with which all patterning steps are monitored during the procedure. After heating the SMP stamp above *T*_g_, which makes the stamp compliant, the mechanical stage with the Si–ODTS–QD substrate is manually raised and brought into conformal contact with the SMP stamp with minimal preload as depicted in Figure 1c. While the preload was applied, the stamp was cooled below *T*_g_ as shown in Figure 1d to induce high pull-off force to remove the QDs from the substrate. Subsequently, the SMP stamp and the Si–ODTS–QD substrate was separated at ~2 µm/s, which removed the QDs on the contact area as schematically shown in Figure 1e.

### 2.5. Characterization

The photoluminescence (PL) spectra of QDs were collected with a Horiba Jobin Yvon FluoroMax-3 spectrofluorometer (Horiba, Ltd., Kyoto, Japan). The scanning electron microscope (SEM) images were obtained on Hitachi S4800 SEM (Hitachi, Ltd., Tokyo, Japan). The thickness of the QD film was measured to be 47 nm using a J. A. Woollam VASE Ellipsometer (J. A. Woollam Co., Inc., Lincoln, NE, USA) in the wavelength range of 400–900 nm at an incidence angle of 60°. Data analysis was performed with WVASE32 software (J. A. Woollam Co., Inc.) using the Cauchy model. PL imaging was carried out on a Jobin Yvon Labram HR800 confocal Raman spectrometer (Horiba, Ltd.) using a 10× air objective with 532 nm laser excitation source. The laser intensity was kept below 0.5 mW to prevent damage to the QD films.

## 3. Results and Discussion

### 3.1. Optical and SEM Images of Patterned QD Films under Different Process Conditions

**Figure 2:**
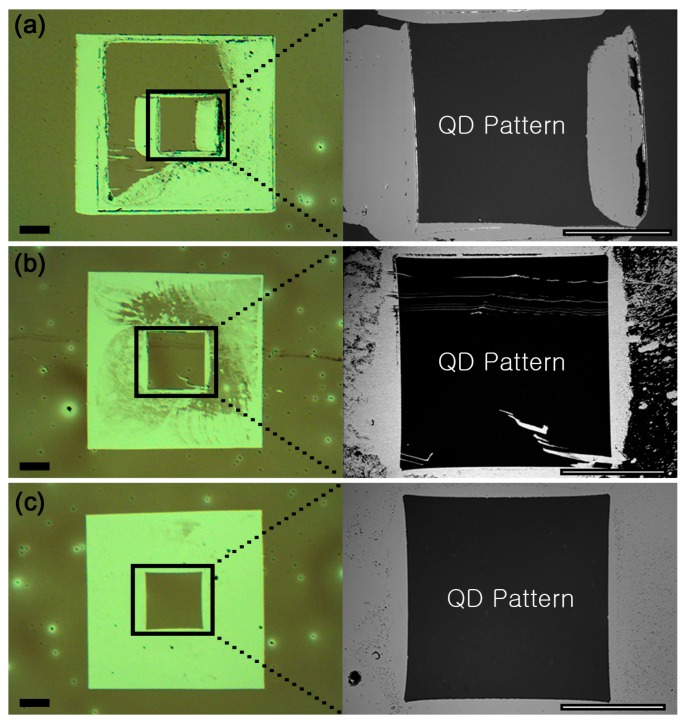
Optical images of the patterned QD films and zoomed in SEM images of the boxed region for different experimental conditions. (**a**) The contacting and separation were both conducted at a temperature below *T*_g_. (**b**) Both contact and separation were conducted at an elevated temperature (above *T*_g_). (**c**) The stamp is heated above *T*_g_ and brought to contact, which is subsequently cooled to room temperature (below *T*_g_) prior to separation. All experiments were conducted at the same preload (~5.5 kPa) and separation rate (~2 µm/s). The scale bars indicate 100 µm.

Optical and scanning electron microscopy (SEM) images of QD films patterned using different states of the SMP stamp with slow separation rate (~2 µm/s) are shown in Figure 2a–c. The result in Figure 2a was achieved with the SMP stamp making contact with the QD substrate below *T*_g_. Owing to the high elastic modulus of the SMP stamp (2.5 GPa [25]) at room temperature (below *T*_g_), the stamp has a very low probability of making highly conformal contact over the entire desired contact area. Any tilting misalignment between the SMP stamp and the QD substrate causes the stamp to slide, leading to an accumulation of QDs. Figure 2b reveals the situation when the SMP stamp is heated at ~80 °C (above *T*_g_) throughout the patterning experiment. Above *T*_g_, the stamp becomes compliant (10 MPa [25]). Relatively well defined square regions suggest conformal contact between the stamp and the QD substrate. However, the pull-off force applied to the QD film during separation is limited by the low elastic modulus, leading to an incomplete removal of the QDs. The effect of changing the elastic modulus of the SMP stamp during QD patterning is shown in Figure 2—in particular, when the stamp is heated during the initial conformal contact and is cooled prior to separation. The patterned region exhibits sharp edges, and the region surrounding the central square pattern has a near complete removal of QDs.

The pull-off force that the SMP stamp exhibits against a rigid surface during separation can be expressed as [29]

(1) $$F_{\mathrm{pull}-\mathrm{off}}=\sqrt{25.31\gamma_{0}\left(2E_{\mathrm{SMP}}\right)L^{3}}$$

where $\gamma_{0}$ is the work of adhesion between the SMP stamp and a rigid surface. $E_{SMP}$ and L are the elastic modulus and the width of the SMP stamp, respectively. Clearly, Equation (1) indicates the pull-off force of the SMP stamp is a function of $E_{\mathrm{SMP}}$ which also depends on whether the SMP stamp is above or below T_g_. Although $\gamma_{0}$ at the SMP-QD interface is unknown and the effect of the elastic moduli mismatch [30] between the SMP and the QDs is simplified, the equation qualitatively shows different pull-off forces of the SMP stamp between above and below T_g_ separation conditions. This difference in pull-off, i.e., separation force, arising from whether or not the stamp is cooled prior to separating the SMP stamp from the QD substrate causes differences in the quality of the SMP stamp patterning of QD films. Because of the high pull-off force afforded by cooling the SMP stamp below T_g_, patterning yield can be completely independent of separation rates.

### 3.2. Characterization of Patterned QD Films

**Figure 3:**
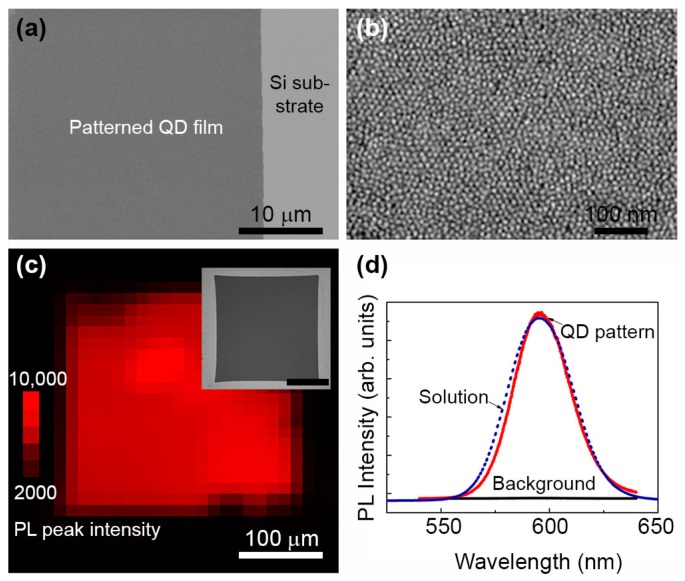
SEM and spatially resolved photoluminescence (PL) of a patterned QD film. (**a**) An SEM image reveals a sharp edge of the patterned QD film. (**b**) Magnified view of the patterned region shows individual QDs uniformly distributed without cracks. (**c**) The PL image of the patterned QD film is consistent with the SEM image shown in the inset. The scale bar in the inset is 100 μm. (**d**) PL spectrum measured within the patterned area of the QD film (solid red curve) is very similar to that measured from a solution of the QDs (dotted blue curve). The slight red-shift of the PL peak of the film state is presumably due to energy transfer between closely packed QDs. The PL from the dark background region (solid black line) surrounding the square pattern of the QD film is significantly reduced.

High magnification SEM images were obtained to characterize the morphology of the patterned QD films. Figure 3a shows a very well-defined edge of the QD pattern using the hot contact and cold separation condition, which corresponds to Figure 2c. Further magnified SEM image of the QD film is shown in Figure 3b. Individual QDs can be seen and are uniformly distributed and densely packed in the film. Additionally, spatially resolved photoluminescence (PL) spectra is obtained to examine the possible effects of the SMP stamp-based patterning process on the optical properties of the QDs. As demonstrated in Figure 3c, the PL intensity mapping matches the SEM image and shows a distinctively different spectrum between the QD pattern and the background. Figure 3d reveals that the PL from the dark regions where QDs have been removed by the SMP stamp is 4 orders of magnitude lower in peak intensity than that from the QD patterns remaining. Furthermore, the PL line shape and peak position are well preserved when compared with those of QDs in solution.

### 3.3. Large Scale Patterning of QD Films

**Figure 4:**
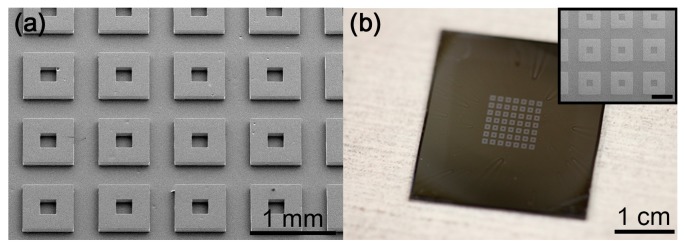
SEM and optical images demonstrating successful large area patterning of QD films utilizing SMP stamps. (**a**) SEM image of the array of SMP stamps and (**b**) optical image of the corresponding patterned QD film. Inset in (**b**) is an SEM image of the patterned QD film with 600 μm scale bar.

The QD patterning achieved here through the SMP stamp is a two-dimensional planar process that can be developed into a large-scale patterning method. In order to demonstrate the feasibility of large scale patterning, an array of 200 µm by 200 µm square SMP stamps is prepared as shown in Figure 4a. The prepared SMP stamp array is brought into contact with a QD substrate at a temperature above *T*_g_ and subsequently cooled below *T*_g_ prior to separation as described in methods. The resulting pattern is shown in Figure 4b. As can be seen in the optical and SEM images, an array of QD patterns covering 1.2 cm × 1.2 cm area can be formed in a simple single patterning step with a low peeling rate using the SMP stamp array.

## 4. Conclusions

This work demonstrates a novel method of patterning QD films under solvent-free, dry conditions. While existing PDMS-based transfer printing approaches can lead to successful QD film patterning, those methods require high peeling rates to induce sufficient pull-off forces. In this context, utilizing the shape memory effect of a SMP that switches its elastic modulus as a function of temperature provides advantages while maintaining desirable solvent-free conditions for patterning QD films. Sufficiently large pull-off forces by the SMP at below *T*_g_ in conjunction with highly conformal contact of the SMP stamp at elevated temperature (above *T*_g_) enable dry patterning of QD films, even at an extremely slow separation rate (2 µm/s), which verifies the rate-independent patterning yield of the SMP stamp. Arrays of QD patterns formed through this method as demonstrated here can facilitate various device applications, such as solar cells, LEDs, and transistors, thereby broadening the applicability of QDs.
